# Prognostic Properties of Instantaneous Amplitudes Maxima of Earth Surface Tremor

**DOI:** 10.3390/e26080710

**Published:** 2024-08-21

**Authors:** Alexey Lyubushin, Eugeny Rodionov

**Affiliations:** Institute of Physics of the Earth RAS, Moscow 123242, Russia; evgeny_980@list.ru

**Keywords:** earth surface tremor, cluster analysis, principal components, Hilbert–Huang decomposition, seismic process, event sequence intensity model

## Abstract

A method is proposed for analyzing the tremor of the earth’s surface, measured by GPS, in order to highlight prognostic effects. The method is applied to the analysis of daily time series of vertical displacements in Japan. The network of 1047 stations is divided into 15 clusters. The Huang Empirical Mode Decomposition (EMD) is applied to the time series of the principal components from the clusters, with subsequent calculation of instantaneous amplitudes using the Hilbert transform. To ensure the stability of estimates of the waveforms of the EMD decomposition, 1000 independent additive realizations of white noise of limited amplitude were averaged before the Hilbert transform. Using a parametric model of the intensities of point processes, we analyze the connections between the instants of sequences of times of the largest local maxima of instantaneous amplitudes, averaged over the number of clusters and the times of earthquakes in the vicinity of Japan with minimum magnitude thresholds of 5.5 for the time interval 2012–2023. It is shown that the sequence of the largest local maxima of instantaneous amplitudes significantly more often precedes the moments of time of earthquakes (roughly speaking, has an “influence”) than the reverse “influence” of earthquakes on the maxima of amplitudes.

## 1. Introduction

This article presents the further development of methods for analyzing ground surface tremor proposed in [1,2,3]. The coherence of the tremor of the earth’s surface was analyzed in [4,5]. In [6,7], coherent analysis of GPS time series was used to assess seismic hazard in Japan and California.

In this case, the main emphasis is on the use of the Hilbert–Huang expansion, which is well suited to take into account the effects of nonstationarity and nonlinearity in time series [8,9]. This method has been successfully used to analyze geodetic time series [10,11], when processing hydrological [12], financial [13], biological [14,15] and seismic [16] data.

The main purpose of the article is to clarify common hypotheses that movements of the earth’s crust recorded by GNSS may contain predictive information. That displacements recorded by geodetic methods respond to the effects of earthquakes is widely known and has been demonstrated many times. But extracting geodetic effects that predict seismic events is much more challenging. In our paper, we propose one method for detecting predictive effects in space geodesy data.

The works [17,18,19,20] analyzed the composition of GPS time series—both their high-frequency part and low-frequency seasonal components—in connection with the estimates of the velocities of tectonic plates. In [21,22,23,24,25,26,27], using multivariate statistical methods, the influence of hydrological loads on tectonic displacements of sections of the earth’s crust was studied. The influence of time delays on the sensitivity of GPS solutions due to the impact of ionospheric and tropospheric factors was analyzed in [28]. The causes of the occurrence of “anomalous harmonics” in the spectral decomposition of GPS time series were considered in [29].

The detailed structure of earth surface displacements presented in GPS time series has been analyzed in the works of a large number of scientists. One of the most popular approaches is the use of the maximum likelihood method for estimating the parameters of GPS time series models [30,31,32,33]. This method was used in [34,35,36] to estimate the parameters of the power spectrum shape and noise amplitude for data from different regions of the world, and the error estimates are discussed in [37,38]. The influence of the spectrum shape and noise amplitude on the errors of displacement velocity estimates was investigated in [39,40]. Phase correlations of GPS time series were studied in [41,42] using parametric models for a number of tectonically active regions.

In [43,44], principal component analysis, empirical orthogonal function analysis, and singular spectrum analysis were used to determine the most common spatial and temporal components of GPS time series. In [45], a joint analysis of accelerometer readings and the noise component of GPS time series was performed. The influence of non-stationary effects on the estimates of relative displacements of crustal blocks and station positions was studied in [46,47,48].

## 2. Data

GPS data of daily earth displacements are taken from the Nevada Geodetic Laboratory website [49] at: http://geodesy.unr.edu/gps_timeseries/tenv3/IGS14/, access at 19 August 2024.

**Figure 1:**
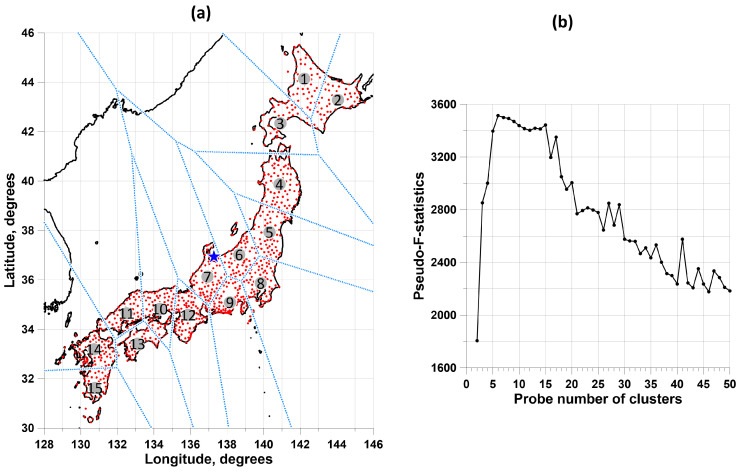
(**a**) shows the positions of 1047 GPS stations and their division into 15 clusters. The numbered circles indicate the centers of gravity of the clusters, and the blue lines indicate the boundaries between Voronoi cells. The blue star shows the position of the center of mass of all cluster centers. (**b**) shows a plot of the pseudo-F-statistic that allowed us to select 15 as the number of clusters.

The set of 1047 GPS stations within domain $30^{\circ }\leq \mathrm{Lat}\leq 46^{\circ }$, $128^{\circ }\leq \mathrm{Lon}\leq 146^{\circ }$, were chosen. These stations presented in Figure 1a have daily time series from the beginning of 2009 to the end of 2023 (15 years), with a total number of gaps of less than 365 samples, and the longest gap less than 182 samples. The vertical components of ground displacement are investigated. The choice of only the vertical component for Japan is due to the fact that it does not contain the catastrophic jump and subsequent long-term relaxation due to the impact of the mega-earthquake of 11 March 2011. Gaps in the GPS time series are filled in using information from the left and right neighborhoods of the gap of the same length as the gap length [4].

The set of stations was previously divided into 15 clusters (Figure 1). The number 15 was chosen as the number of clusters, which optimally splits their “cloud”. Let us split the set of station position vectors $\vec{\zeta }$ into a given number q of clusters using the k-means clustering method [50]. Let $C_{r},\;r=1,\ldots ,q$ be clusters, let ${\vec{z}}_{r}=\sum_{\vec{\zeta }\in \;C_{r}}\vec{\zeta }/n_{r}$ be the vector of the center of mass of the cluster $C_{r}$, and let $n_{r}$ be the number of vectors in the cluster, $\sum_{r=1}^{q}n_{r}=N$. Vector $\vec{\zeta }\in C_{r}$ if the distance $\left|\vec{\zeta }-{\vec{z}}_{r}\right|$ is the minimum among the positions of all cluster centers. The k-means method minimizes the sum of squared distances:

(1) $$G({\vec{z}}_{1},\ldots ,{\vec{z}}_{q})=\sum_{r=1}^{q}\sum_{\vec{\zeta }\in C_{r}}{\left|\vec{\zeta }-{\vec{z}}_{r}\right|}^{2}\to \underset{{\vec{z}}_{1},\ldots ,{\vec{z}}_{q}}{\min}$$

relative to the position of the cluster centers ${\vec{z}}_{r}$. Let $\Phi (q)=\underset{{\vec{z}}_{1},\ldots ,{\vec{z}}_{q}}{\min}G({\vec{z}}_{1},\ldots ,{\vec{z}}_{q})$. We used a trial number of clusters in the range 2≤q≤50. The problem of choosing the best number $q^{*}$ of clusters was solved using the maximum pseudo-F-statistic criterion [51]

(2) $$PFS(q)=\sigma_{1}^{2}(q)/\sigma_{0}^{2}(q)\to \underset{2\;\leq \;q\;\leq \;50}{\max}$$

where

(3) $$\sigma_{0}^{2}(q)=\frac{\Phi (q)}{N-q},\;\sigma_{1}^{2}(q)=\sum_{r=1}^{q}\frac{n_{r}}{N}\cdot {\left|{\vec{z}}_{r}-{\vec{z}}_{0}\right|}^{2},\;{\vec{z}}_{0}=\frac{1}{N}\sum_{1}^{N}\vec{\zeta }$$

The plot in Figure 1b presents the pseudo-F-statistic values as a function of the trial number of clusters. The number 15 on the pseudo-F-statistics graph is the inflection point of the dependence on the trial number of clusters and realizes one of the largest local maxima for the number of clusters from 2 to 50. On the pseudo-F-statistics graph, they represent two local maxima with close values of the number of clusters 6 and 15. Of these two values, 15 was chosen as the largest in order to provide the most detailed breakdown of the set of stations. Figure 1a shows the division of a set of stations into 15 clusters along with Voronoi cells, which indicate that the stations belong to a particular cluster.

Clusters of stations are ordered by increasing latitude of the position of their centers of gravity. Table 1 shows for each cluster (first row) the number of stations in the cluster (second row).

## 3. Principal Components of Increments in a Moving Time Window

Since the goal is to study the tremor of the earth’s surface, that is, the high-frequency part of the earth’s surface displacements, the analysis was carried out for increments of time series. Switching to increments reduces the dominant influence of low frequencies in the daily GPS time series and ensures stationarity of time series fragments within the 365-day time windows that are used further.

The division of a set of stations into 15 clusters is used for the subsequent application of the principal component method [52]. For each cluster of stations, the first principal component of the time series of increments of vertical displacements of the earth’s surface was calculated in a sliding adaptation time window of 365 days in length.

Let there be a *p*-dimensional cloud of similar *N*-dimensional signals $\{y_{j}(k),\;k=1,\ldots ,N\},\;j=1,\ldots ,p$. Let us choose the size of the sliding window w and center the signals,

(4) $$x_{j}(k)=y_{j}(k)-\;\bar{y_{j}(k)},\;j=1,\ldots ,p,\;k=w,\ldots ,N,$$

(5) $$\bar{y_{j}(k)}=\frac{1}{w}\sum_{i=1}^{w}y_{j}(k-w+i),\;k=w,\ldots ,N.$$

The next step is to calculate the sample estimate of the covariance (p×p)-dimensional matrix in a sliding window:

(6) $$r_{XX}^{\left(m,n\right)}=\frac{1}{w}\sum_{i=1}^{w}x_{m}(i-w+k)x_{n}(i-w+k),\;k,j=1,\ldots ,p,\;k=w,\ldots ,N.$$

Let $\phi^{\left(s\right)}={\left(\phi_{1}^{\left(s\right)},\ldots ,\phi_{p}^{\left(s\right)}\right)}^{T}$ be the eigenvector of this matrix corresponding to the maximum eigenvalue. Let us put

(7) $$\xi_{s}(k)\;=\;\sum_{j=1}^{p}{\left(\phi_{j}^{\left(k\right)}\right)}^{2}x_{j}(k),\;k=w,\ldots ,N.$$

Having generalized Formulas (4)–(7) with understandable changes to the case k=1,…,w−1, let us determine the weighted average in a sliding time window of length w using the formula

(8) $$\xi (k)=\left\{ \begin{array}{l} \xi_{w-1}(k),\;k<w,\\ \xi_{k}(k),\;k\geq w. \end{array}\right.\;$$

Thus, Formulas (7) and (8) determine the values of the weighted average increments of vertical time series of displacements of the earth’s surface. The squared values of the eigenvector of the covariance matrix in the sliding time window corresponding to the largest eigenvalue are taken as weights. The sum of these weights is equal to one.

Within each of the 15 clusters, a transition to a weighted average was made using the method described above; the length w of the sliding time window was taken as equal to 365 samples, i.e., 1 year. At the same time, in order to eliminate the influence of large outliers, before calculating the weighted average, the so-called winsorization procedure was carried out [53], which consists in eliminating outliers falling beyond the level μ±4σ by cutting off the values of the time series in a sliding time window (μ and σ are sample estimates of the mathematical expectation and standard deviation for the current time window). The procedure is repeated iteratively until the values μ and σ stop changing.

**Figure 2:**
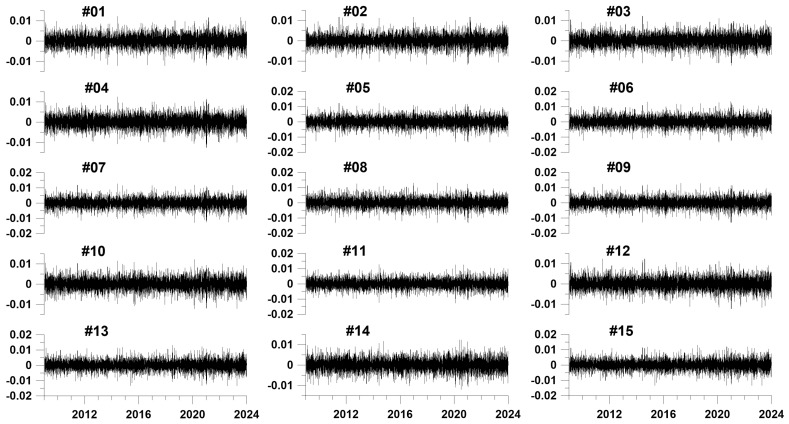
Graphs of weighted average vertical displacements of the earth’s surface in each of the selected 15 clusters in a sliding time window of 365 days. The Y axes represent displacement increments in mm.

Figure 2 shows graphs of the first principal components of increments (in the form of weighted averages) of vertical displacements of the earth’s surface in each of the identified 15 clusters.

## 4. Empirical Mode Decomposition

Let x(k) be the analyzed discrete signal. Empirical mode decomposition (EMD) [8,9] represents the decomposition of the signal into modes of oscillation:

(9) $$x(k)=\sum_{j=1}^{n}h_{j}(k)+r_{n}(k)$$

where $h_{j}(k)$ is the *j*-th empirical mode, $r_{n}(k)$ is the remainder, *n* is the number of empirical modes.

The algorithm for decomposing into a sequence of empirical modes is iterative for each level j. Let us denote by $m,\;m=0,1,\ldots ,M_{j}$ the index of iterations, where $M_{j}$ is the maximum number of iterations for level j. The iterations are described by the formula

(10) $$h_{j}^{\left(m+1\right)}(k)=h_{j}^{\left(m\right)}(k)-z_{j}^{\left(m\right)}(k),$$

Here $z_{j}^{\left(m\right)}(k)=\left(p_{j}^{\left(m\right)}(k)+q_{j}^{\left(m\right)}(k)\right)/2$, where $p_{j}^{\left(m\right)}(k)$ and $q_{j}^{\left(m\right)}(k)$ are both the upper and lower envelopes for the signal, which are constructed using spline interpolation (usually a third order spline) over all local maxima and minima of the signal $h_{j}^{\left(m\right)}(k)$.

Iterations (10) are initialized with step zero for the first level (j=1) of the expansion $h_{1}^{\left(0\right)}(k)=x(k)$. Next, the upper $p_{1}^{\left(0\right)}(k)$ and lower $q_{1}^{\left(0\right)}(k)$ envelopes are found, and the middle line $z_{1}^{\left(0\right)}(k)$ is calculated and found $h_{1}^{\left(2\right)}(k)$ using Formula (10). For $h_{1}^{\left(1\right)}(k)$, the upper $p_{1}^{\left(1\right)}(k)$ and lower $q_{1}^{\left(1\right)}(k)$ envelopes are determined, and the middle line $z_{1}^{\left(1\right)}(k)$ is found, and so on, until the last iteration index $M_{1}$, after which it is considered that the first empirical mode $h_{1}(k)$ has been found.

The condition for stopping iterations is usually chosen in the form of the following inequality:

(11) $$\sum_{k}{\left(h_{j}^{\left(m+1\right)}(k)-h_{j}^{\left(m\right)}(k)\right)}^{2}/\sum_{k}{\left(h_{j}^{\left(m\right)}(k)\right)}^{2}\;\leq \;\delta ,$$

where δ is some small number, for example 0.01. After the mode $h_{j}(k)$ is found, the iterative process of determining the empirical mode $h_{j+1}(k)$ of the next level is started. This process is initialized by the formula for the initial iteration index m=0:

(12) $$h_{j+1}^{\left(0\right)}(k)=x(k)-h_{j}(k),$$

According to formula (12), the high-frequency part is subtracted from the original signal and a new, lower-frequency signal is considered as a new signal for subsequent decomposition. The construction of empirical oscillation modes continues until the number of local extrema becomes too small for envelopes to be constructed from them. As the number j of the empirical mode level increases, the signals become increasingly low-frequency and tend towards an unchanging form. The sequence $h_{1}(k),\;h_{2}(k),\ldots ,h_{n}(k)$ is constructed in such a way that its sum gives an approximation to the original signal x(k), which can be represented in formula (9) [8,9]. Empirical modes are orthogonal to each other, thus constituting a certain empirical basis for the decomposition of the original signal.

In the practical implementation of the method, technical difficulties arise due to edge effects, since the continuation of the envelopes beyond the first and last points of local extrema is ambiguous. To overcome this difficulty, there are several approaches, in particular mirror continuation [8,13] of the analyzed sample back and forth for a sufficiently long period of time. It was this approach that was used in this work.

## 5. Ensemble Empirical Mode Decomposition

One of the key disadvantages of the EMD method is the problem of mode mixing, which occurs when one empirical mode includes signals of different scales or when signals of the same scale are distributed over different empirical modes. For example, if “intermittency” is observed in the signal, that is, against the background of a smooth signal, short-term sections of higher frequency behavior appear, then with EMD decomposition behavior modes with different frequencies are mixed, since relatively rare points of local extrema of smooth behavior are interspersed with much more frequent points of local extrema of the high-frequency component.

To combat this effect, ref. [9] proposed the ensemble empirical mode decomposition (EEMD) method. This is a regularization of the EMD method in which white noise of finite amplitude is added to the original data. This allows the true empirical modes to be determined as the average over an ensemble of trials, each of which is the sum of signal and white noise.

The EEMD algorithm includes the following steps:

1. Add a white noise implementation to the original data.
2. Decomposition of data with the addition of white noise into empirical modes.
3. Repeat steps 1 and 2 quite a large number of times with different implementations of white noise.
4. Obtain the ensemble average for the corresponding empirical modes.

Thus, numerous “artificial” observations are simulated:

(13) $$x^{\left(i\right)}(k)=x(k)+\varepsilon_{i}(k),$$

where $\varepsilon_{i}(k)$ is the *i*-th realization of white noise.

The true component, according to the EEMD definition, for a sequence of all levels of empirical modes is calculated as the average value of the expansions of the noisy modes (13). It is important to note that EEMD largely eliminates this mixing problem [9]. Adding independent white noise to the sample has a regularizing effect, since it simplifies the construction of envelopes (after adding a small amount of white noise, many local extrema are immediately created). The operation of averaging over a sufficiently large number of independent implementations of white noise makes it possible to eliminate the influence of the noise component and to isolate the true internal modes of oscillations.

**Figure 3:**
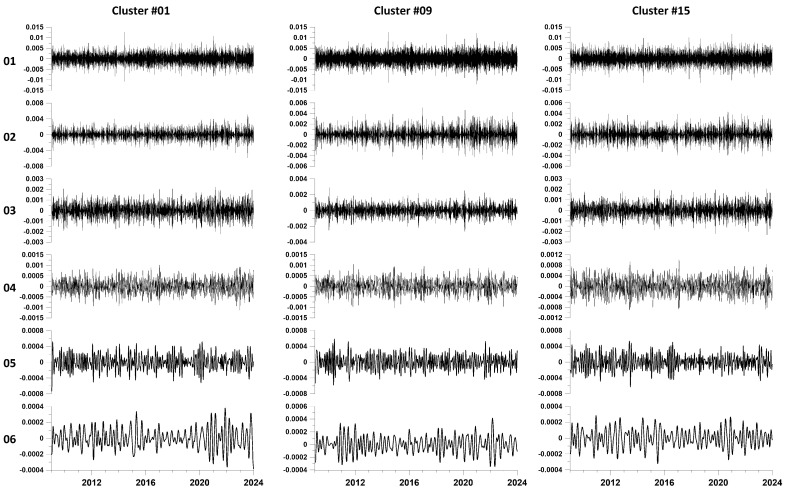
EEMD waveform plots for the first 6 decomposition levels for 3 clusters (numbers 1, 9 and 15). Decomposition level numbers are indicated on the left.

For each of the resulting 15 time series, EEMD waveforms of principal components were calculated. EEMD waveforms are obtained by averaging 1000 decompositions of the original signals, to which are added independent Gaussian white noises with a standard deviation of 0.1 from the standard deviation of the weighted average from each cluster. Figure 3 shows EEMD waveform plots for the first 6 expansion levels for three of the 15 clusters.

## 6. Hilbert Transform

The Hilbert transform of the signal is determined by the formula [54]:

(14) $$H_{X}(t)=\int_{-\infty }^{+\infty }\frac{X(u)}{\pi (t-u)}\;du=X(t)*\left(\frac{1}{\pi t}\right)$$

where $f(t)*g(t)=\int_{-\infty }^{+\infty }f(u)\cdot g(t-u)\;du$ is the convolution of two functions. If $\tilde{f}(\omega )$ and $\tilde{g}(\omega )$ are the Fourier transforms of convolutional functions $\tilde{f}(\omega )=\int_{-\infty }^{+\infty }f(t)\cdot e^{-i\omega t}\;dt$, then, as is known, the Fourier transform of convolution is equal to the product of the Fourier transforms of convolutional functions. Fourier transform from 1/(πt) equals:

(15) $$\int_{-\infty }^{+\infty }\frac{e^{-i\omega t}}{\pi t}\;dt=-i\cdot sign(\omega )=\{\begin{array}{c} -i,\;\omega >0\\ 0,\;\omega =0\\ i,\;\omega <0 \end{array}$$

Thus, if $\tilde{X}(\omega )$ there is a Fourier transform of X(t), then

(16) $${\tilde{H}}_{X}(\omega )=-i\cdot sign(\omega )\cdot \tilde{X}(\omega )=\{\begin{array}{c} -i\cdot \tilde{X}(\omega ),\;\omega >0\\ 0,\;\;\;\;\omega =0\\ i\cdot \tilde{X}(\omega ),\;\omega <0 \end{array}$$

If you present $\tilde{X}(\omega )=|\tilde{X}(\omega )|e^{-i\varphi (\omega )}$, then

(17) $${\tilde{H}}_{X}(\omega )=\{\begin{array}{c} |\tilde{X}(\omega )|e^{-i(\varphi (\omega )+\pi /2)},\;\omega >0\\ 0,\;\;\;\;\;\;\;\omega =0\\ |\tilde{X}(\omega )|e^{-i(\varphi (\omega )-\pi /2)},\;\omega <0 \end{array}$$

In practice, it is more convenient to calculate the Hilbert transform using the concepts of an analytical signal:

(18) $$Z_{X}(t)=X(t)+i\cdot H_{X}(t)=|Z_{X}(t)|\cdot e^{i\vartheta (t)}\equiv A_{X}(t)\cdot e^{i\vartheta (t)}$$

where $A_{X}(t)=\sqrt{X^{2}(t)+H_{X}^{2}(t)}$ are the amplitudes of the signal X(t) envelope, and ϑ(t) is the instantaneous phase. The derivative ν(t)=dϑ(t)/dt is called instantaneous frequency. The Fourier transform of the analytical signal ${\tilde{Z}}_{X}(\omega )=\tilde{X}(\omega )+i\cdot {\tilde{H}}_{X}(\omega )=\tilde{X}(\omega )\cdot (1+sign(\omega ))$ or:

(19) $${\tilde{Z}}_{X}(\omega )=\{\begin{array}{c} 2\tilde{X}(\omega ),\;\omega >0\\ \tilde{X}(0),\;\;\omega =0\\ 0,\;\;\;\;\;\omega <0 \end{array}$$

after which the Hilbert transform is equal to the imaginary part of the result of the inverse Fourier transform of ${\tilde{Z}}_{X}(\omega )$

(20) HX(t)=Im(12π∫−∞+∞Z˜X(ω)eiωtdω)

**Figure 4:**
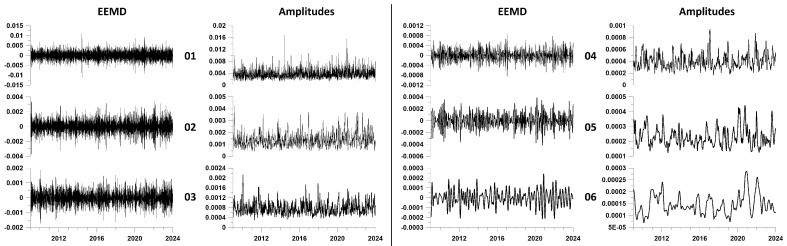
Plots of mean EEMD waveforms and mean instantaneous amplitudes averaged over all 15 station clusters for the first 6 decomposition levels. The left side of the figure, separated by a vertical blue line, shows pairs of graphs (waveforms—their amplitudes) for levels 1–3, and the right side shows graphs for levels 4–6. The decomposition level numbers are indicated between the waveform and amplitude graphs.

For each of the resulting 15 time series, EEMD waveforms of principal components were calculated. EEMD waveforms are obtained by averaging 1000 decompositions of the original signals, to which are added independent Gaussian white noises with a standard deviation of 0.1 from the standard deviation of the weighted average from each cluster. Figure 4 shows EEMD waveform plots for the first 6 expansion levels for three of the 15 clusters.

For a discrete-time signal X(t), t=0,…,(N−1), this transformation can be calculated using the discrete Fourier transform:

(21) $$d_{X}^{\left(N\right)}(\omega_{k})=\sum_{t=0}^{N-1}X(t)\cdot \exp(-i\omega_{k}t),\;\omega_{k}=\frac{2\pi }{N}(k-1),\;k=0,1,\ldots ,(N-1)$$

after which the second part of the Fourier coefficients (corresponding to negative frequencies) should be reset to zero: $h_{X}^{\left(N\right)}(\omega_{k})=0,\;k=N/2+1,\ldots ,(N-1)$ while the 1st part should be doubled: $h_{X}^{\left(N\right)}(\omega_{k})=\;2\cdot d_{X}^{\left(N\right)}(\omega_{k}),\;k=1,\ldots ,N/2$. The Hilbert transform is then calculated as the imaginary part of the inverse discrete Fourier transform:

(22) HX(t)=Im (∑k=0N−1h X(N)(ωk)⋅exp(iωkt)/N), t=0,1,…,(N−1)

Thus, after determining the instantaneous amplitudes and frequencies of the EEMD, the Hilbert–Huang decomposition can be represented as follows:

(23) x(t)=∑j=1nhj(t)+rn(t), hj(t)=Re {A j(t)⋅exp(i⋅∫νj(s) ds)}

## 7. Influence Matrix

To solve the problem of estimating the connection between two sequences of random events, a parametric intensity model is used. In [55,56,57] this method was used to test the hypotheses that local extrema of the average values of certain properties of seismic noise and magnetic field precede the instants of strong earthquakes.

Let $t_{j}^{\left(\alpha \right)},\;j=1,\ldots ,N_{\alpha };\;\alpha =1,2$ represent the moments in time of 2 streams of events.

In our case it is:

(1) a sequence of moments in time corresponding to the largest local maxima of the amplitudes of the envelopes at certain levels of the EEMD Huang decomposition
(2) a sequence of times of seismic events with a magnitude not less than a given value.

Let us present their intensities in the form:

(24) $$\lambda^{\left(\alpha \right)}(t)=b_{0}^{\left(\alpha \right)}\;+\;\sum_{\beta =1}^{2}b_{\beta }^{\left(\alpha \right)}\cdot g^{\left(\beta \right)}(t)$$

where $b_{0}^{\left(\alpha \right)}\geq 0,b_{\beta }^{\left(\alpha \right)}\geq 0$ are parameters, and $g^{\left(\beta \right)}(t)$ is the influence function of flow events $t_{j}^{\left(\beta \right)}$ with number β:

(25) $$g^{\left(\beta \right)}(t)=\sum_{t_{j}^{\left(\beta \right)}<t}\exp(-(t-t_{j}^{\left(\beta \right)})/\tau )$$

According to formula (25), the weight of an event with number j becomes non-zero for times $t\;>\;t_{j}^{\left(\beta \right)}$ and decays with a characteristic time τ. The parameter $b_{\beta }^{\left(\alpha \right)}$ determines the degree of influence of the sequence β on the sequence α. The parameter $b_{\alpha }^{\left(\alpha \right)}$ determines the degree of self-excitation to which the flow α influences itself, and the parameter $b_{0}^{\left(\alpha \right)}$ reflects the purely random (Poisson) intensity component. Let us fix the parameter τ and consider the problem of estimating the parameters $b_{0}^{\left(\alpha \right)},b_{\beta }^{\left(\alpha \right)}$.

The log-likelihood function for a non-stationary Poisson process within the time interval [0,T] equals [58]:

(26) $$\ln(L_{\alpha })=\sum_{j=1}^{N_{\alpha }}\ln(\lambda^{\left(\alpha \right)}(t_{j}^{\left(\alpha \right)}))-\int_{0}^{T}\lambda^{\left(\alpha \right)}(s)ds,\;\alpha =1,2$$

We need to find parameters $b_{0}^{\left(\alpha \right)},b_{\beta }^{\left(\alpha \right)}$ from maximum of functions (26). It is easy to obtain the following expression:

(27) $$b_{0}^{\left(\alpha \right)}\frac{\partial \ln(L_{\alpha })}{\partial b_{0}^{\left(\alpha \right)}}+\sum_{\beta =1}^{2}b_{\beta }^{\left(\alpha \right)}\frac{\partial \ln(L_{\alpha })}{\partial b_{\beta }^{\left(\alpha \right)}}=N_{\alpha }-\int_{0}^{T}\lambda^{\left(\alpha \right)}(s)ds$$

The parameters $b_{0}^{\left(\alpha \right)},b_{\beta }^{\left(\alpha \right)}$ are non-negative. It means that each term on the left side of (27) equals to zero at point of maximum of function (26)—either due to the necessary conditions for the extremum (if the parameters are positive), or, if the maximum is reached at the boundary, then the parameters themselves are equal to zero. Therefore, at the maximum point of the likelihood function the equality holds:

(28) $$\int_{0}^{T}\lambda^{\left(\alpha \right)}(s)ds=N_{\alpha }$$

Let us substitute the expression $g^{\left(\beta \right)}(t)$ from (28) into (27) and divide by T. Then we get another form of formula (28):

(29) $$b_{0}^{\left(\alpha \right)}+\sum_{\beta =1}^{m}b_{\beta }^{\left(\alpha \right)}\cdot {\bar{g}}^{\left(\beta \right)}=\lambda_{0}^{\left(\alpha \right)}\equiv N_{\alpha }/T$$

where

(30) $${\bar{g}}^{\left(\beta \right)}=\int_{0}^{T}g^{\left(\beta \right)}(s)ds/T$$

average value of the influence function. Substituting $b_{0}^{\left(\alpha \right)}$ from (29) into (26), we obtain the following maximum problem:

(31) $$\Phi^{\left(\alpha \right)}(b_{1}^{\left(\alpha \right)},b_{2}^{\left(\alpha \right)})=\sum_{j=1}^{N_{\alpha }}\ln(\lambda_{0}^{\left(\alpha \right)}+\sum_{\beta =1}^{2}b_{\beta }^{\left(\alpha \right)}\cdot \Delta g^{\left(\beta \right)}(t_{j}^{\left(\alpha \right)}))\to \max$$

where $\Delta g^{\left(\beta \right)}(t)=g^{\left(\beta \right)}(t)-{\bar{g}}^{\left(\beta \right)}$, under restrictions:

(32) $$b_{1}^{\left(\alpha \right)}\geq 0,b_{2}^{\left(\alpha \right)}\geq 0,\sum_{\beta =1}^{2}b_{\beta }^{\left(\alpha \right)}{\bar{g}}^{\left(\beta \right)}\leq \lambda_{0}^{\left(\alpha \right)}$$

It could be shown that function (31) is convex with a negative definite Hessian. Therefore, problem (31) and (32) has a unique solution. The problem (31) and (32) is solved numerically for a given relaxation parameter τ. After this step we can define the influence matrix with elements $\kappa_{\beta }^{\left(\alpha \right)},\alpha =1,2;\;\beta =0,1,2$ using the formulas:

(33) $$\kappa_{0}^{\left(\alpha \right)}=\frac{b_{0}^{\left(\alpha \right)}}{\lambda_{0}^{\left(\alpha \right)}}\geq 0,\;\kappa_{\beta }^{\left(\alpha \right)}=\frac{b_{\beta }^{\left(\alpha \right)}\cdot {\bar{g}}^{\left(\beta \right)}}{\lambda_{0}^{\left(\alpha \right)}}\geq 0$$

The value $\kappa_{0}^{\left(\alpha \right)}$ is the share of the average intensity $\lambda_{0}^{\left(\alpha \right)}$ of the process with number α, which is purely random, part $\kappa_{\alpha }^{\left(\alpha \right)}$ is caused by the influence of self-excitation α→α, and $\kappa_{\beta }^{\left(\alpha \right)},\;\beta \neq \alpha$ is due to external influence β→α. From Formula (29) the normalization condition follows:

(34) $$\kappa_{0}^{\left(\alpha \right)}+\sum_{\beta =1}^{2}\kappa_{\beta }^{\left(\alpha \right)}=1,\;\alpha =1,2$$

As a result, the influence matrix can be determined:

(35) $$\left(\left|\begin{array}{l} \kappa_{0}^{\left(1\right)}\\ \kappa_{0}^{\left(2\right)} \end{array}\right|\left|\begin{array}{l} \kappa_{1}^{\left(1\right)}\;\kappa_{2}^{\left(1\right)}\\ \kappa_{1}^{\left(2\right)}\;\kappa_{2}^{\left(2\right)} \end{array}\right|\right)$$

The first column of matrix (35) is composed of Poisson shares of average intensities. The diagonal elements of the right submatrix of size 2 × 2 consist of self-exciting elements of mean intensity, while the off-diagonal elements correspond to mutual excitation. The sums of the component rows of the influence matrix (34) equal 1. The influence matrices are estimated in a certain moving time window of length L with an offset ΔL and with a given value of the relaxation parameter τ.

## 8. Estimation of Connections between the Times of Local Amplitude Maxima and Seismic Events

**Figure 5:**
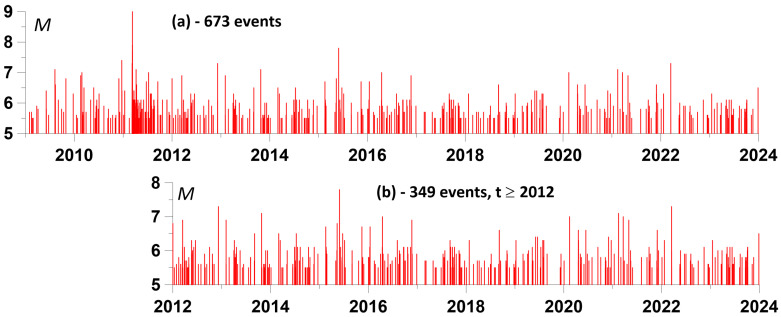
Time sequence of earthquakes with a magnitude of at least 5.5 in the vicinity of the Japanese Islands: (**a**) in the time period 2009–2023; (**b**) in the period of time 2012–2023.

The further plan of the article is to use the apparatus of influence matrices to assess the relationship between the times of maximum average amplitudes of the envelopes and the times of sufficiently strong earthquakes. A magnitude threshold of 5.5 was chosen. There were 673 such seismic events in the vicinity of the Japanese islands during the period of time 2009–2023—see Figure 5a. However, the mega-earthquake of 11 March 2011 with a magnitude of 9 gave rise to a surge in aftershock activity, as a result of which, if we consider the time interval 2012–2023, when the intensity of aftershocks had already decreased, the number of earthquakes with a magnitude of at least 5.5 will decrease by almost 2 times to 349—see Figure 5b. It should be noted that accurately estimating the time of the end of the Tohoku earthquake aftershocks is a difficult task [59] and in this case we used a rough estimate based on the visual perception of the intensity of seismic events.

The working hypothesis is that for certain levels of EEMD decomposition, the times of the largest local maxima of the average amplitudes of the envelopes precedes the times of earthquakes. For a correct comparison of two streams of events, it is necessary that their average intensity be approximately equal. This means that the number of the largest local maxima of the amplitudes of the envelopes during the time period under study should be equal to the number of earthquakes. From these considerations, it becomes clear that for a correct analysis of the connections between the time instants of local maximum amplitudes of envelopes and seismic events, the time interval of aftershock activity must be excluded. Therefore, further analysis is carried out for the time period 2012–2023 lasting 12 years.

**Figure 6:**
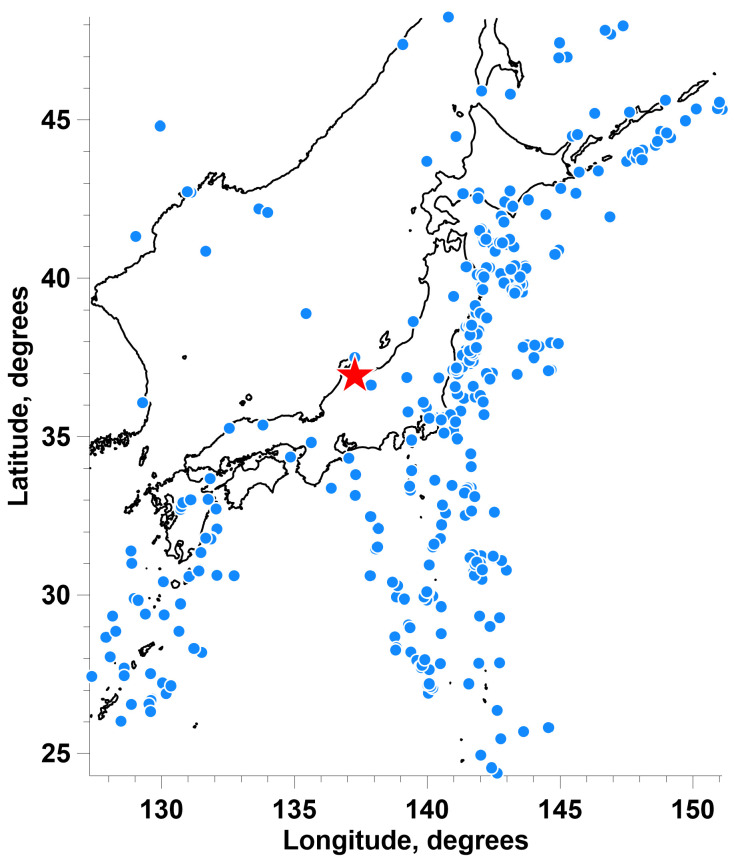
Distribution of epicenters of 349 earthquakes with a magnitude of at least 5.5 in the vicinity of the Japanese Islands in the time period 2012–2023. The red asterisk marks the center of gravity of the centers of 15 clusters of GPS stations (Figure 1a), which is chosen as the center of a circle with a radius of 1500 km.

Figure 6 shows the distribution of epicenters of earthquakes with a magnitude of at least 5.5.

It should be taken into account that as the number of the decomposition level increases, both the waveforms of the levels themselves and the amplitudes of their envelopes become increasingly low-frequency. As a result, it is possible to select the 349 largest local maxima of average amplitudes in the interval 2012–2023 only for a certain number of lower expansion levels. For the time interval 2012–2023, only the first two levels are suitable for selecting 349 local maxima of amplitudes, since the number of local extrema already at the third level is 242, that is, less than 349. From the first two levels, it was decided to choose the second as the lower frequency, and for which the results of mutual influence assessments are more expressive.

**Figure 7:**
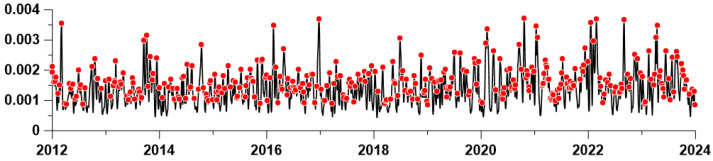
Average instantaneous amplitudes of the second level of EEMD decomposition and the 349 largest local maxima (red dots).

In Figure 7, the red dots represent the selected 349 largest local maxima of the average amplitude of the envelopes at the second level of decomposition.

**Figure 8:**
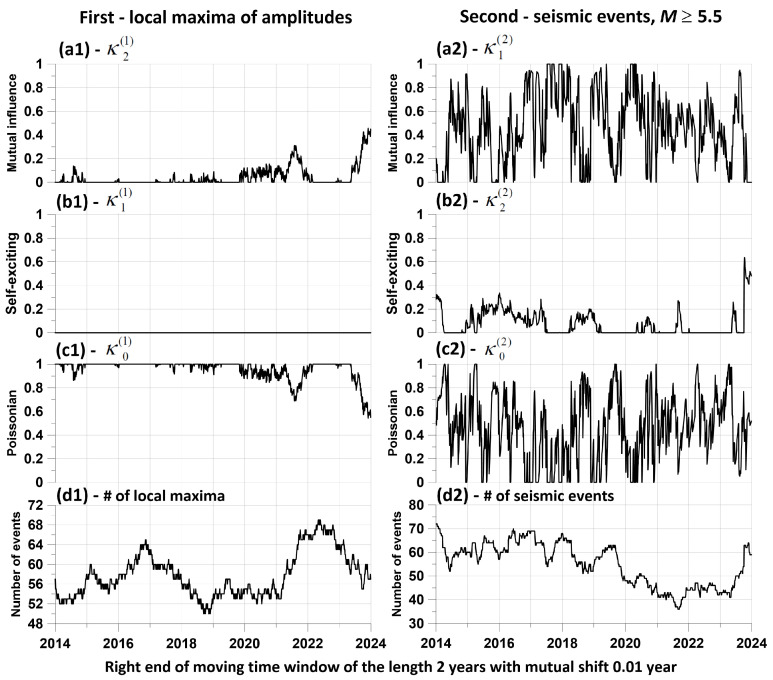
Graphs of changes in the components of the influence matrix between sequences of seismic events with a magnitude of at least 5.5 and the sequence of moments in time of 349 of the largest local maxima of the average amplitudes at the second level of EEMD decomposition. The estimates were made in a sliding time window of length 2 years with a shift of 0.01 year for a relaxation time τ of the model (24, 25) of 0.1 year. The graphs (**a1**–**c1**) refer to the components of the influence matrix (35), which refers to the intensity fractions of the sequence of the largest local amplitude maxima, while the graphs (**a2**–**c2**) refer to the intensity fractions of the sequence of seismic events. Plots (**d1**) and (**d2**) present the numbers of local maxima of amplitudes (**d1**) and the number of seismic events (**d2**) within moving time window. Other explanations are in the text.

Let us call the “direct” influence of the moments of time of earthquakes on the occurrence of local maxima of the average amplitudes of the envelopes, and the “reverse” —correspondingly, the advance of the moments of time of local maxima of amplitudes relative to the times of earthquakes. Figure 8 shows graphs of changes in the components of the matrix of “direct” and “reverse” influence for level 2 when assessed in a sliding window of 2 years.

Of these graphs, the pair (a1, a2) is of greatest importance: a1 represents the change in the components of the direct influence of seismic events on the positions of local amplitude maxima, while a2 represents the inverse influence of the times of local amplitude maxima on seismic events. From a comparison of these two graphs, it is clear that the reverse influence significantly exceeds the direct influence; that is, there is a delay effect of seismic events relative to the maximum amplitudes. In other words, there is a predictive effect. Graphs (b1, b2) represent changes in the self-exciting component of average intensities, while graphs (c1, c2) represent changes in the purely random (Poisson) component. Finally, the pair of plots (d1, d2) represents the change in the number of jointly analyzed time points in each time window. Let us recall once again that the sum of the components (a1, b1, c1) and (a2, b2, c2) is equal to 1 for any position of the time window.

Figure 8 presents the results of estimates of the mutual influence of two sequences of events only for a time window of 2 years. In order to increase the representativeness of this result, we will carry out similar estimates for a sufficiently large set of time lengths varying within specified limits. In this case, for each value of the length of the time window, we will identify local maxima of the components of the influence matrix, which are responsible for the mutual influence of sequences of events when the time windows are shifted. Let us describe a method for constructing a set of maximum components of mutual influence matrices in the form of numbered points.

1. The minimum $L_{\min}$ and maximum $L_{\max}$ lengths of time windows and $N_{L}$—the number of lengths of time windows in this interval are selected. Thus, the lengths of the time windows took on the values $L_{k}=L_{\min}+(k-1)\Delta L$, $k=1,\ldots ,N_{L}$, $\Delta L=(L_{\max}-L_{\min})/(N_{L}-1)$. In our calculations, we took $L_{\min}$ as equal to 1 year, and $L_{\max}$—3 years, $N_{L}=100$.
2. Each time window of length $L_{k}$ was shifted from left to right along the time axis with some offset Δt. Let us denote by $t_{j}(L_{k})$, $j=1,\ldots ,M(L_{k})$ the sequence of moments in time of the positions of the right windows with length $L_{k}$. The number $M(L_{k})$ of time windows in length $L_{k}$ is determined by their time offset Δt. We used a time window offset Δt of 0.01 year.
3. For each position of a time window of length $L_{k}$, the elements of the influence matrix (35) are estimated for a given relaxation time τ of the model (26–27), corresponding to the mutual influence of the two processes being analyzed. We took a value τ equal to 0.1 year. For definiteness, we will consider one influence, for example, of the first process on the second. As a result of such estimates, we obtain their values in the form $\left(t_{j}(L_{k}),\;c_{j}(L_{k})\right)$, where $c_{j}(L_{k})$ is the corresponding element of the influence matrix for a position with a time window number j of length $L_{k}$.
4. In the sequence $\left(t_{j}(L_{k}),\;c_{j}(L_{k})\right)$, we select elements $\left(t_{j}^{*}(L_{k}),\;c_{j}^{*}(L_{k})\right)$ corresponding to local maxima of values $c_{j}(L_{k})$, that is, from the condition $c_{j-1}(L_{k})<c_{j}^{*}(L_{k})<c_{j+1}(L_{k})$. Let us present each element $\left(t_{j}^{*}(L_{k}),\;c_{j}^{*}(L_{k})\right)$ as a vertical segment of length $c_{j}^{*}(L_{k})$ located at a time point $t_{j}^{*}(L_{k})$. The combination of such vertical graphic elements for all $k=1,\ldots ,N_{L}$, $j=1,\ldots ,M(L_{k})$ visualizes the “strength” of the mutual influence of processes on each other.

**Figure 9:**
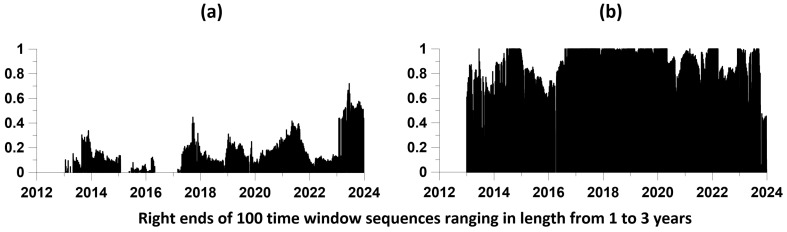
Maximum values of the elements of the influence matrices in a sequence of 100 time windows of length from 1 to 3 years, taken with an offset of 0.01 year: (**a**) for the “direct” influence of the time points of seismic events on the positions of the largest local maxima of average amplitudes on the second EEMD level of decomposition; (**b**) for the “reverse” influence of the positions of amplitude maxima on earthquakes. The relaxation time of the model is 0.1 year.

So, the full set of free parameters of the method: τ, $L_{\min}$, $L_{\max}$, $N_{L}$, Δt. The result of such estimates is presented in Figure 9.

The results presented in Figure 9 confirm the conclusions made earlier based on the graphs in Figure 8: the “reverse” influence of the time instants of local maxima of envelope amplitudes at the second level on the time instants of earthquakes is significantly greater (Figure 9b) than the “direct” influence of earthquakes on the occurrence of local maxima in the amplitudes of the envelopes (Figure 9a).

## 9. Conclusions

Traditional methods of analyzing data on crustal movements obtained using space geodesy are focused on identifying systematic low-frequency components that can be interpreted as manifestations of slow tectonic movements. The high-frequency component of these time series, which can be called the “tremor” of the earth’s surface, is most often interpreted as a manifestation of noise arising from atmospheric and ionospheric fluctuations. Our point is that, despite the presence of this process noise, it is in the high-frequency component of GPS data that there is hidden prognostic information. In this article, the joint use of cluster analysis, principal component analysis, Hilbert–Huang decomposition and evaluation of parametric models of the mutual influence of sequences of events allowed us to obtain a result confirming the presence of predictive information in high-frequency tremor of the earth’s surface.

The authors used software which was written by them using programming languages Fortran and Python.

## Figures and Tables

**Table 1:** Number of N_sta_ stations in each Clust# cluster.

Clust#.	1	2	3	4	5	6	7	8	9	10	11	12	13	14	15
N_sta_	57	56	54	83	69	61	78	77	91	76	57	95	48	88	57

## Data Availability

The open access data from the source: http://geodesy.unr.edu/gps_timeseries/tenv3/IGS14/ (accessed on 15 January 2024) were used.
